# Quantifying cover crop effects on soil health and productivity

**DOI:** 10.1016/j.dib.2020.105376

**Published:** 2020-03-05

**Authors:** Jinshi Jian, Xuan Du, Ryan D. Stewart

**Affiliations:** a School of Plant and Environmental Sciences, Virginia Tech, 330 Smyth Hall, 185 Ag Quad Lane, Blacksburg, VA 24061, USA; b Joint Global Change Research Institute, Pacific Northwest National Laboratory, 5825 University Research Ct. #3500, College Park, MD, USA; c Department of Hydraulic Engineering, Yangling Vocational & Technical College, Yangling, Shaanxi, China

**Keywords:** Soil health, Soil quality, Cover crop, Conservation management, Agriculture

## Abstract

The dataset presented here supports the research paper entitled “A calculator to quantify cover crop effects on soil health and productivity”. Soil health (sometimes used synonymously with soil quality) is a concept that describes soil as a living system to sustain plants, animals, and human. Soil physical, chemical, and biological properties, along with their interactions, are required to quantify soil health. The use of cover crops in agricultural rotations may enhance soil health, yet there has been little progress in understanding how external factors such as climate, soil type, and agronomic practices affect soil and cash crop responses. In response, this dataset compiles measurements from 281 studies and provides an analysis of field-measured changes in 38 soil health indicators due to cover crop usage. Environmental and background indicators were also compiled to assess how climatic and management practices affect soil and cash crop responses to cover crops, with specific categories including climate type (tropical, arid, temperate, and continental), soil texture (coarse, medium, and fine), cover crop type (legume, grass, multi-species mixture, and other), and cash crop type (corn, soybean, wheat, vegetable, corn-soybean rotation, corn-soybean-wheat rotation, and other). An unbalanced analysis of variation was used to determine the hierarchy of most to least important factors that affected responsiveness of each soil health indicator. Based on the hierarchy structure, a soil health calculator was then developed to quantify the response of 13 parameters – erosion, runoff, weed suppression, soil aggregate stability, leaching, infiltration, microbial biomass carbon, soil bulk density, soil organic carbon, soil nitrogen, microbial biomass nitrogen, cash crop yield, and saturated hydraulic conductivity – to cover crops. The presented data in the calculator report the mean change in parameter values based on all combinations of climate, soil texture, cover crop type, and cash crop type.

**Table:** **Specification Table**

Subject	Soil science, agronomy
Specific subject area	Soil degradation, agricultural sustainable development, soil health, crop yield, soil erosion, nutrient leaching
Type of data	Table Figure Supplemental table R code
How data were acquired	Systematic literature search, data extraction, data filtration with quality control, data analysis
Data format	Raw Analyzed and processed Filtered
Parameters for data collection	Using search term “soil health” or “soil quality” and “conservation management” or “cover crop” in ISI Web of Science, Google Scholar, and the China National Knowledge Infrastructure (CNKI).
Description of data collection	Data from 281 articles were extracted and compiled into the dataset. Data were either directly read from tables or figures. When reading data from figures, we used the software Data Thief (version III, https://datathief.org/).
Data source location	Global, but with majority data from middle latitude, especially North America, Europe, and China.
Data accessibility	Repository name: [SoilHealthCalculator] Direct URL to data: https://github.com/jinshijian/SoilHealthCalculator.
Related research article	Jian, J., B. J. Lester, X. Du, M. S. Reiter, and R. D. Stewart (2020). A calculator to quantify cover crop effects on soil health and productivity. Soil & Tillage Research 199, 104575 [1].

## Value of the Data

• The dataset quantifies cover crop effects on soil physical, chemical, and biological properties.
• This dataset supports future systematic reviews and meta-analyses of soil health.
• This dataset supports a global soil health calculator.
• Farmers and extension agents can benefit from this dataset when accessing the web-based soil health calculator presented in the companion article.

## 1 Data description

The dataset here summarizes the data processing and analysis to support the creation of a web-based soil health calculator [1]. The data were compiled from a global soil health database (*SoilHealthDB*) [2,3], and represent 281 published articles from 38 countries.

*Supplemental Document* 1 includes the full list of references and describes the meta-information of 281 published articles used in this dataset.

*Supplemental Document* 2 describes the UANOVA analysis outputs of all 38 indicators.

*Supplemental Document* 3 describes the hierarchy structure that identifies the most to least important factor (i.e., hierarchical layer) for 13 indicators (for details regarding these indicators please see Table 1 in the related research article [1]).

*Supplemental Document* 4 summarizes the data used to develop a web-based soil health calculator.

**Fig. 1:**
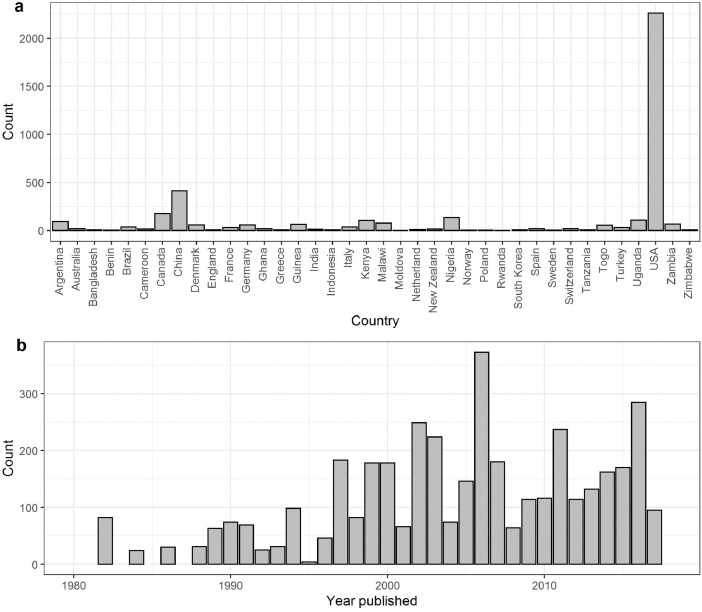
Number of records for each country (a) and number of records published per year (b). Note that two studies published before 1980 were omitted in panel b.

Fig. 1 describes the number of records from 38 countries and the number of records published through year.

**Fig. 2:**
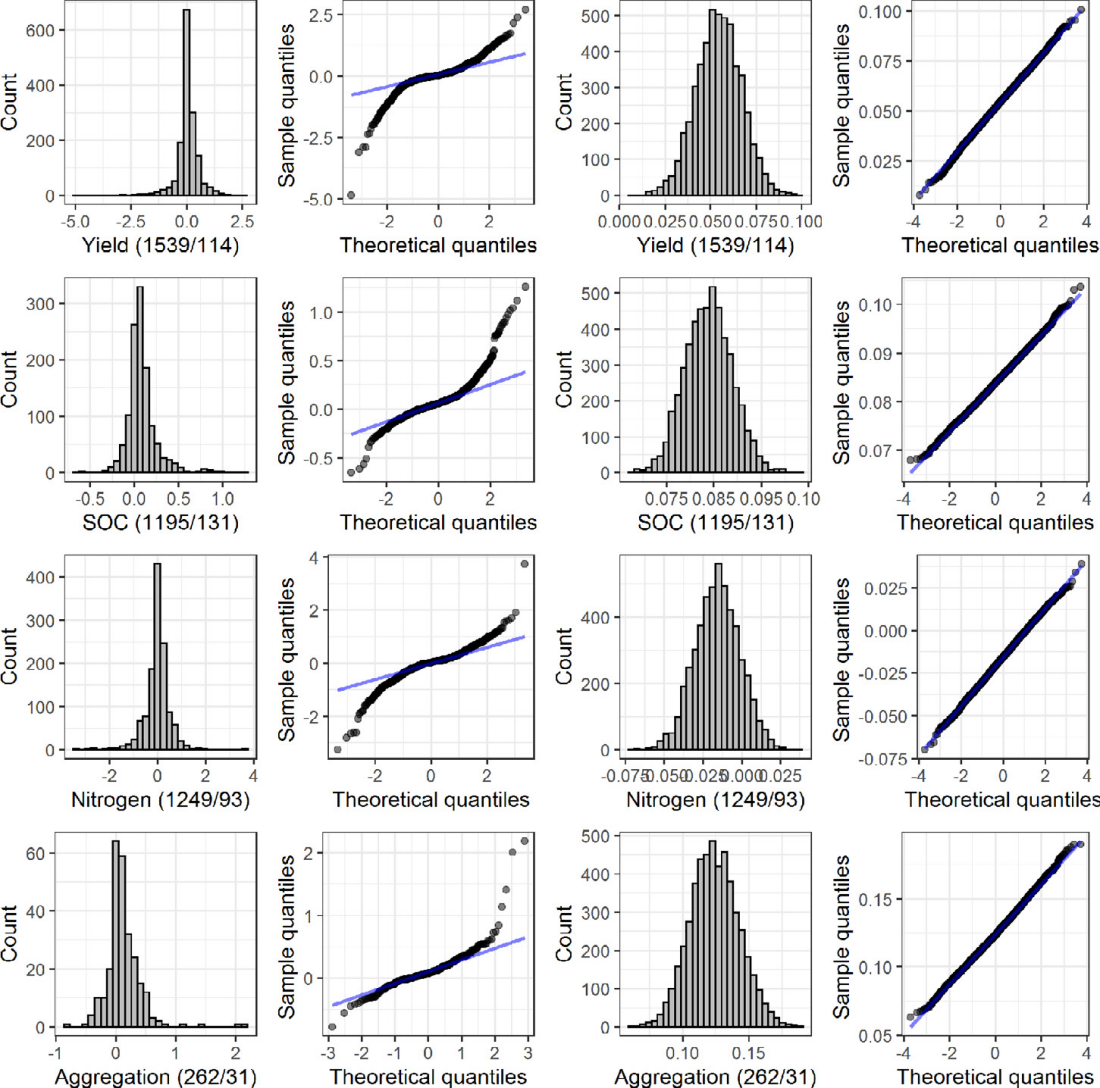
Histogram of log transformed ratio (RR) and the theoretical quantiles vs. sample quantiles (Q-Q) for yield, SOC, Nitrogen, and Aggregation. The two columns on the left show raw data, while the two columns on the right show resampled data using bootstrapping. Note that the numbers in the parenthesis represent number of observations and number of studies (e.g., yield had 1539 observations from 114 studies). Outputs for other indicators are found at https://github.com/jinshijian/SoilHealthCalculator.

Fig. 2 shows the histogram and theoretical quantiles vs. sample quantiles (Q-Q) of log transformed response ratio (RR) for yield, SOC, Nitrogen, and Aggregation (left two columns show raw data, while right two columns show resampled data using bootstrapping).

**Fig. 3:**
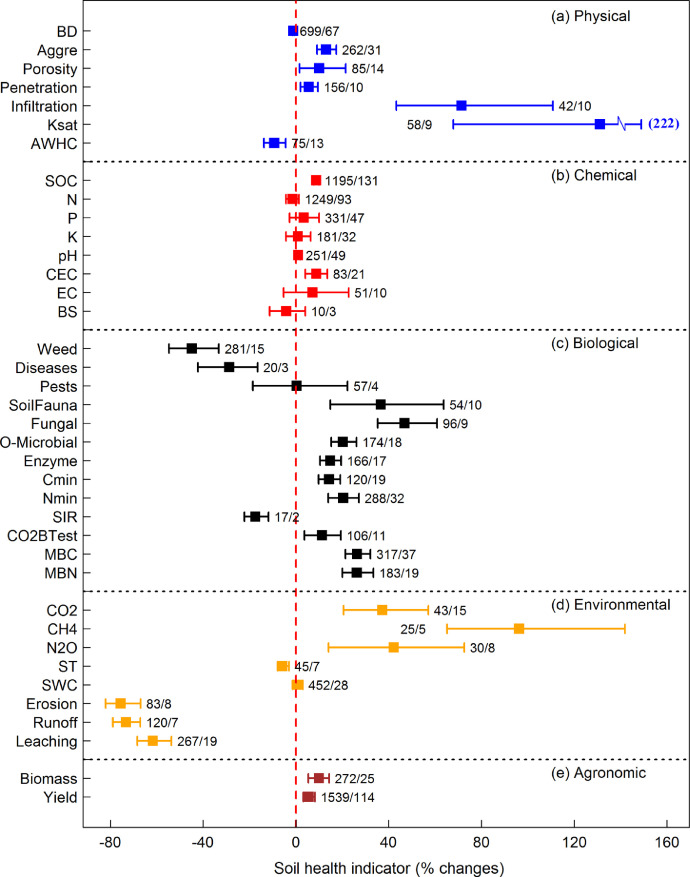
Bootstrapping outputs comparing cover crops and control values for 38 soil health parameters. Whiskers indicate the 95% confidence intervals for each set of response ratios, scaled to % change.

Fig. 3 describes the bootstrapping outputs of RR (in percent changes) for 38 soil health parameters (whiskers indicate the 95% confidence intervals). Note that the bootstrapping outputs were comparable with yet distinct from the reults presented in the related research article (Fig. 3; [1]), which instead showed data from one sample t-tests.

**Fig. 4:**
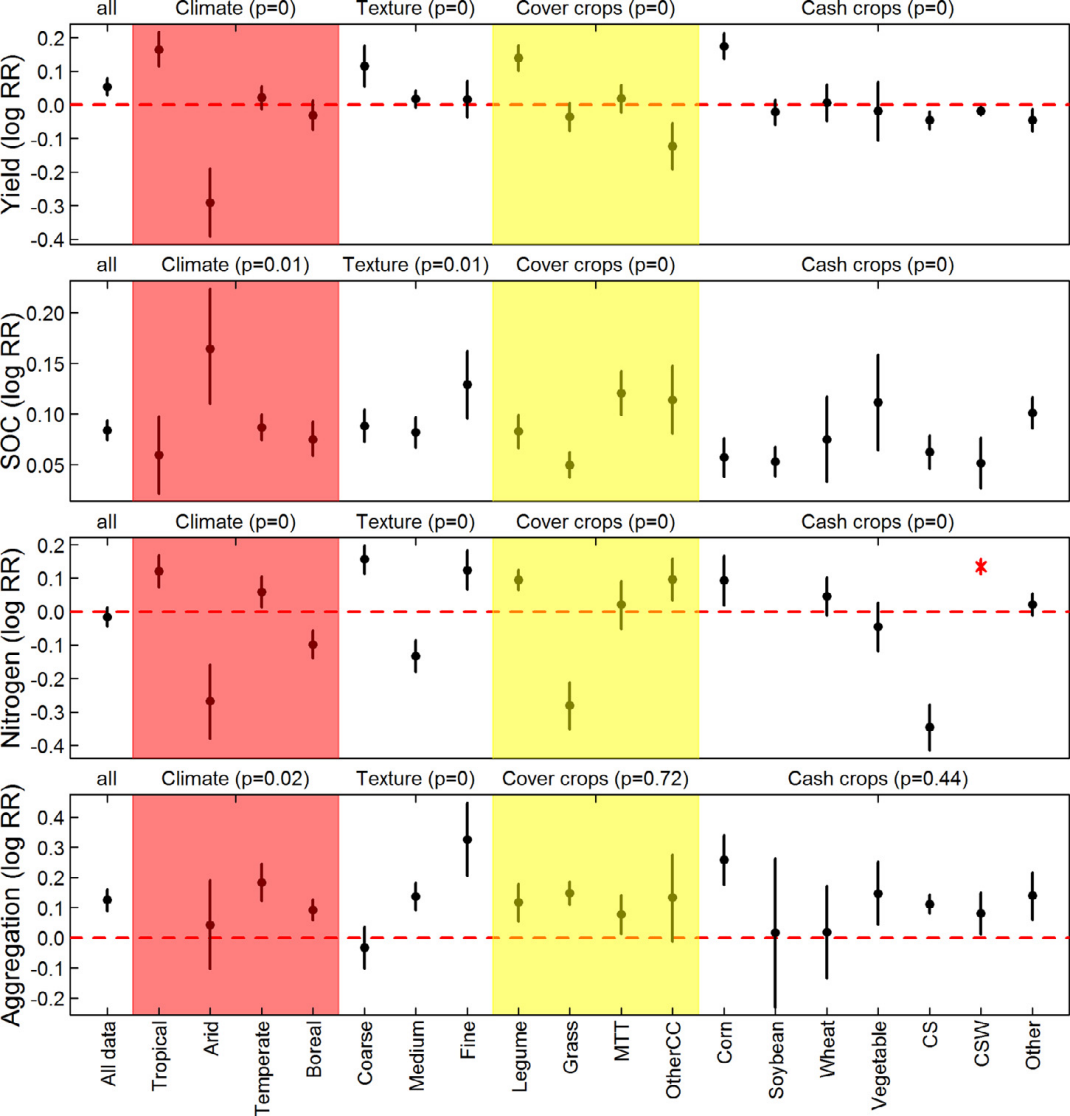
Mean and 95% confidence interval of log transformed response ratio of yield, SOC, Nitrogen, and Aggregation, based on climate type, soil texture, cover crop type, and cash crop type. Note that the p-values in the parenthesis represent outputs for unbalanced ANOVA. The dot and error bar in red indicates < 3 samples; outputs for other indicators are presented in the GitHub repository (https://github.com/jinshijian/SoilHealthCalculator).

Fig. 4 describes the mean and 95% confidence interval of RR (log transformed) for yield, SOC, Nitrogen, and Aggregation, based on climate type, soil texture, cover crop type, and cash crop type.

**Fig. 5:**
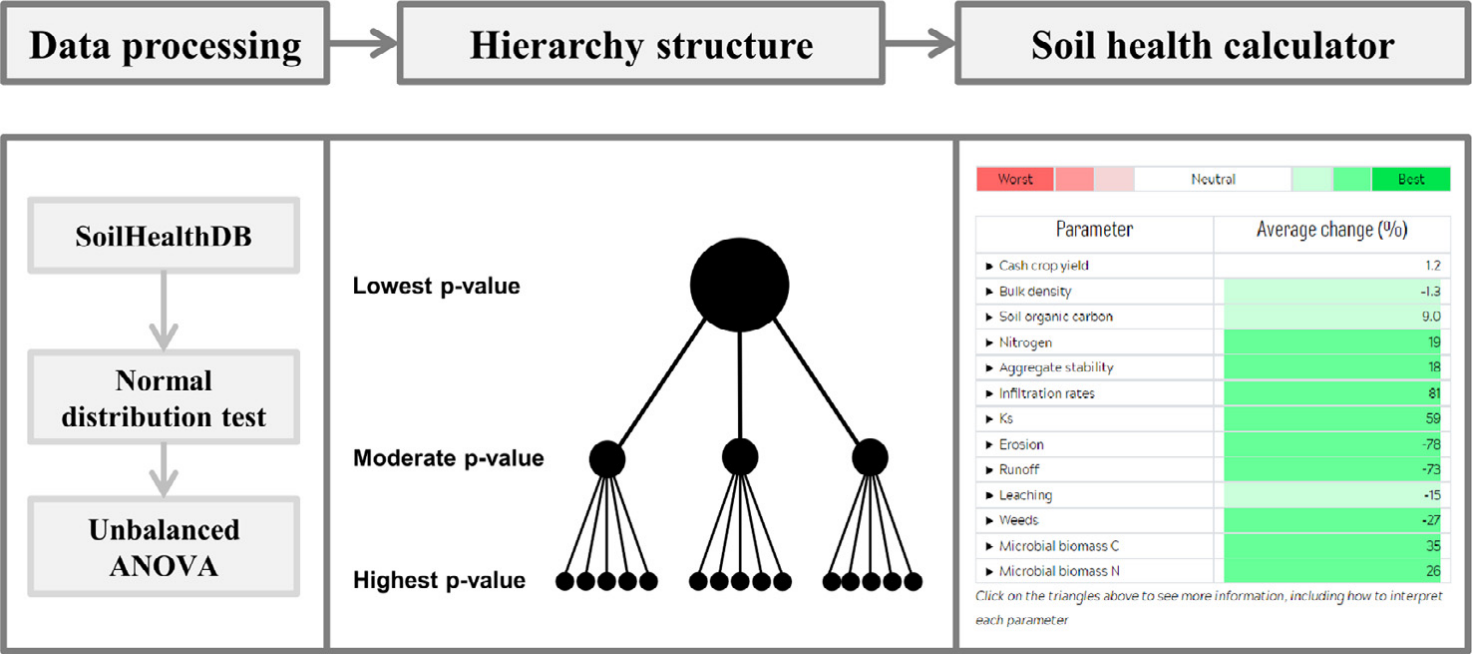
Overview of the data processing procedure, hierarchical layer structure, and soil health calculator output showing average % change for 13 indicators.

Fig. 5 shows the data processing procedure, hierarchical layer structure, and soil health calculator output, which presents average % change for 13 indicators (for details of these 13 indicators please see Table 1 in the related research article [1]).

## 2 Experimental design, materials, and methods

We quantified the response ratio for each observation of a soil health indicator as RR = ln(X_cc_/X_nc_), where X_cc_ indicates the parameter value in the cover crop treatment and X_nc_ represents the parameter value in the no cover crop control. All RR values for a given indicator were assembled. To ensure normality, RR distributions were resampled (with replacement) 1000 times via bootstrapping (Fig. 2). Mean values and 95% confidence intervals were then estimated from the resampled distributions for each parameter (Fig. 3).

We next used an unbalanced analysis of variation (UANOVA) to test for significant differences in RR values in 38 soil health indicators, examining the data as grouped by Koppen climate type (tropical, arid, temperate, or continental [4]), soil texture (coarse, medium, or fine [5,6]), cover crop type (legume, grass, multi-species mixture, or other), and cash crop type (corn, soybean, wheat, vegetable, corn-soybean rotation, corn-soybean-wheat rotation, or other). Examples of the UANOVA analysis are presented in Fig. 4, with the full analysis provided in *Supplemental Document* 2.

We next developed a hierarchy structure that identifies the most to least important factor (i.e., hierarchical layer) for each parameter. Climate was set as the highest level, while the remaining three factors were ordered from lowest to highest p-values in the unbalanced ANOVA. This analysis was applied to calculate mean RR for all combinations of climate type, soil texture, cash crop type, and cover crop type, specifically focusing on 13 key soil health indicators: cash crop yield, bulk density, soil organic carbon, soil nitrogen, soil aggregation, soil infiltration, soil saturated conductivity, soil erosion, surface runoff, leaching, weed pressures, soil microbial carbon, and soil microbial nitrogen (*Supplemental Document* 3). As the final step, these data were used to develop a web-based soil health calculator (Fig. 5), with the underlying data compiled in *Supplemental Document* 4.

## Appendix Supplementary materials

Image, application 1
