# Supplementary material for: Quantifying cover crop effects on soil health and productivity
Source: Data Brief. 2020 Mar 5;29:105376. doi: 10.1016/j.dib.2020.105376 (PMC7078303; doi:10.1016/j.dib.2020.105376)
Supplement: Supplementary file 1 [file mmc1.zip › Supplemental_document_1_Article_List.docx]

**Supplemental document 1.** Studies compiled in this dataset.

1. Abawi, G. S. & Widmer, T. L. Impact of soil health management practices on soilborne pathogens, nematodes and root diseases of vegetable crops. Appl. Soil Ecol. 15, 37–47 (2000).

2. Bandick, A. K. & Dick, R. P. Field management effects on soil enzyme activities. Soil Biol. Biochem. 31, 1471–1479 (1999).

3. Blackshaw, R. E. Agronomic merits of cereal cover crops in dry bean production systems in western Canada. Crop Prot. 27, 208–214 (2008).

4. Blanco-Canqui, H., Mikha, M. M., Presley, D. R. & Claassen, M. M. Addition of Cover Crops Enhances No-Till Potential for Improving Soil Physical Properties. Soil Sci. Soc. Am. J. 75, 1471 (2011).

5. Blanco-Canqui, H., Holman, J. D., Schlegel, A. J., Tatarko, J. & Shaver, T. M. Replacing Fallow with Cover Crops in a Semiarid Soil: Effects on Soil Properties. Soil Sci. Soc. Am. J. 77, 1026 (2013).

6. Bloszies, S. A. Soil Microbial Activity and Organic Carbon Dynamics in Low Input Agroecosystems. (North Carolina State University, 2016).

7. Bottomley, P. J., Sawyer, T. E., Boersma, L., Dick, R. P. & Hemphill, D. D. Winter cover crop enhances 2,4-D mineralization potential of surface and subsurface soil. Soil Biol. Biochem. 31, 849–857 (1999).

8. Brainard, D. C., Bryant, A., Noyes, D. C., Haramoto, E. R. & Szendrei, Z. Evaluating pest-regulating services under conservation agriculture: A case study in snap beans. Agric. Ecosyst. Environ. 235, 142–154 (2016).

9. Bruce, R. R., Langdale, G. W., West, L. T. & Miller, W. P. Soil surface modification by biomass inputs affecting rainfall infiltration. Soil Sci. Soc. Am. J. 56, 1614–1620 (1992).

10. Bulan, M. T. S., Stoltenberg, D. E. & Posner, J. L. Buckwheat species as summer cover crops for weed suppression in no-tillage vegetable cropping systems. Weed Sci. 63, 690–702 (2015).

11. Buyer, J. S., Teasdale, J. R., Roberts, D. P., Zasada, I. A. & Maul, J. E. Factors affecting soil microbial community structure in tomato cropping systems. Soil Biol. Biochem. 42, 831–841 (2010).

12. Campbell, R. B., Sojka, R. E. & Karlen, D. L. Conservation tillage for soybean in the US southeastern cosstal plain. Soil Tillage Res. 4, 531–541 (1984).

13. Campbell, C. A., Biederbeck, V. O., Zentner, R. P. & Lafond, G. P. Effect of crop rotations and cultural practices on soil organic matter, microbial biomass and respiration in a thin Black Chernozem. Can. J. Plant Sci. 71, 363–376 (1991).

14. Carr, P. M., Anderson, R. L., Lawley, Y. E., Miller, P. R. & Zwinger, S. F. Organic zero-till in the northern US Great Plains Region: Opportunities and obstacles. Renew. Agric. Food Syst. 27, 12–20 (2012).

15. Duiker, S. W. & Curran, W. S. Rye cover crop management for corn production in the Northern mid-Atlantic region. Agron. J. 97, 1413–1418 (2005).

16. Ess, D. R., Vaughan, D. H. & Perumpral, J. V. Crop residue and root effects on soil compaction. Trans. ASAE 41, 1271–1275 (1998).

17. Finney, D. M., White, C. M. & Kaye, J. P. Biomass production and carbon/nitrogen ratio influence ecosystem services from cover crop mixtures. Agron. J. 108, 39–52 (2016).

18. Finney, D. M., Buyer, J. S. & Kaye, J. P. Living cover crops have immediate impacts on soil microbial community structure and function. J. Soil Water Conserv. 72, 361–373 (2017).

19. Hargrove, W. L. Winter Legumes as a Nitrogen Source for No-Till Grain Sorghum. Agron. J. 78, 70–74 (1986).

20. Hayden, Z. D., Ngouajio, M. & Brainard, D. C. Rye-vetch mixture proportion tradeoffs: Cover crop productivity, nitrogen accumulation, and weed suppression. Agron. J. 106, 904–914 (2014).

21. Hinds, J. & Hooks, C. R. R. Population dynamics of arthropods in a sunn-hemp zucchini interplanting system. Crop Prot. 53, 6–12 (2013).

22. Hinds, J., Wang, K.-H. K.-H., Marahatta, S. P., Meyer, S. L. F. & Hooks, C. R. R. Sunn hemp cover cropping and organic fertilizer effects on the nematode community under temperate growing conditions. J. Nematol. 45, 265–271 (2013).

23. Hinds, J., Wang, K. H. & Hooks, C. R. R. Growth and yield of zucchini squash (Cucurbita pepo L.) as influenced by a sunn hemp living mulch. Biol. Agric. Hortic. 32, 21–33 (2016).

24. Hubbard, R. K., Strickland, T. C. & Phatak, S. Effects of cover crop systems on soil physical properties and carbon/nitrogen relationships in the coastal plain of southeastern USA. Soil Tillage Res. 126, 276–283 (2013).

25. Idowu, O. J. et al. Use of an integrative soil health test for evaluation of soil management impacts. Renew. Agric. Food Syst. 24, 214–224 (2009).

26. Jani, A. D., Grossman, J., Smyth, T. J. & Hu, S. Winter legume cover-crop root decomposition and N release dynamics under disking and roller-crimping termination approaches. Renew. Agric. Food Syst. 31, 214–229 (2016).

27. Jernigan, A. B. et al. Weed abundance and community composition following a long-Term organic vegetable cropping systems experiment. Weed Sci. 65, 639–649 (2017).

28. Jiang, P., Anderson, S. H., Kitchen, N. R., Sadler, E. J. & Sudduth, K. a. Landscape and conservation management effects on hydraulic Properties of a claypan-soil toposequence. Soil Sci. Soc. Am. J. 71, 803 (2007).

29. Jokela, W. E., Grabber, J. H., Karlen, D. L., Balser, T. C. & Palmquist, D. E. Cover crop and liquid manure effects on soil quality indicators in a corn silage system. Agron. J. 101, 727–737 (2009).

30. Joyce, B. A. et al. Infiltration and soil water storage under winter cover cropping in California’s Sacramento Valley. Trans. ASAE 45, 315–326 (2002).

31. Kabir, Z. & Koide, R. T. The effects of dandelion or a cover crop onmycorrhizal inoculum potential, soil aggregation and yield of maize. Agric. Ecosyst. Environ. 78, 167–174 (2000).

32. Kaspar, T. C., Jaynes, D. B., Parkin, T. B. & Moorman, T. B. Rye Cover Crop and Gamagrass Strip Effects on NO Concentration and Load in Tile Drainage. J. Environ. Qual. 36, 1503 (2007).

33. Keene, C. L. & Curran, W. S. Optimizing high-residue cultivation timing and frequency in reduced-tillage soybean and corn. Agron. J. 108, 1897–1906 (2016).

34. Kladivko, E. J. et al. Nitrate Leaching to Subsurface Drains as Affected by Drain Spacing and Changes in Crop Production System. J. Environ. Qual. 33, 1803 (2004).

35. Kong, A. Y. Y., Scow, K. M., Córdova-Kreylos, A. L., Holmes, W. E. & Six, J. Microbial community composition and carbon cycling within soil microenvironments of conventional, low-input, and organic cropping systems. Soil Biol. Biochem. 43, 20–30 (2011).

36. Langdale, G. W., West, L. T., Bruce, R. R., Miller, W. P. & Thomas, A. W. Restoration of eroded soil with conservation tillage. Soil Technol. 5, 81–90 (1992).

37. Lehman, R. M., Taheri, W. I., Osborne, S. L., Buyer, J. S. & Douds, D. D. Fall cover cropping can increase arbuscular mycorrhizae in soils supporting intensive agricultural production. Appl. Soil Ecol. 61, 300–304 (2012).

38. Liebig, M., Carpenter-Boggs, L., Johnson, J. M. F., Wright, S. & Barbour, N. Cropping system effects on soil biological characteristics in the Great Plains. Renew. Agric. Food Syst. 21, 36–48 (2007).

39. Marriott, E. E. & Wander, M. Qualitative and quantitative differences in particulate organic matter fractions in organic and conventional farming systems. Soil Biol. Biochem. 38, 1527–1536 (2006).

40. Mbuthia, L. W. et al. Long term tillage, cover crop, and fertilization effects on microbial community structure, activity: Implications for soil quality. Soil Biol. Biochem. 89, 24–34 (2015).

41. McCracken, D. V., Smith, M. S., Grove, J. H., Blevins, R. L. & MacKown, C. T. Nitrate Leaching as Influenced by Cover Cropping and Nitrogen Source. Soil Sci. Soc. Am. J. 58, 1476 (1994).

42. McVay, K. A., Radcliffe, D. E. & Hargrove, W. L. Winter Legume Effects on Soil Properties and Nitrogen Fertilizer Requirements. Soil Sci. Soc. Am. J. 53, 1856 (1989).

43. Mendes, I. C., Bandick, A. K., Dick, R. P. & Bottomley, P. J. Microbial biomass and activities in soil aggregates affected by winter cover crops. Soil Sci. Soc. Am. J. 63, 873–881 (1999).

44. Ndiaye, E. L., Sandeno, J. M., McGrath, D. & Dick, R. P. Integrative biological indicators for detecting change in soil quality. Am. J. Altern. Agric. 15, 26 (2000).

45. Nielsen, D. C. & Vigil, M. F. Legume green fallow effect on soil water content at wheat planting and wheat yield. Agron. J. 97, 684–689 (2005).

46. O’Dea, J. K., Miller, P. R. & Jones, C. A. Greening summer fallow with legume green manures: On-farm assessment in north-central Montana. J. Soil Water Conserv. 68, 270–282 (2013).

47. Osborne, S. L. et al. The Impact of Corn Residue Removal on Soil Aggregates and Particulate Organic Matter. Bioenergy Res. 7, 559–567 (2014).

48. Quinn, N. F., Brainard, D. C. & Szendrei, Z. The Effect of Conservation Tillage and Cover Crop Residue on Beneficial Arthropods and Weed Seed Predation in Acorn Squash. Environ. Entomol. 45, 1543–1551 (2016).

49. Epth, T. I. D., Iming, T. I. T., Rop, C. O. C. & On, E. F. Tillage depth, tillage timing, and cover crop effects on cotton yield, soil strength, and tillage energy requirements. Appl. Eng. Agric. 16, 379–385 (2000).

50. Rasse, D. P., Ritchie, J. T., Peterson, W. R., Wei, J. & Smucker, A. J. M. Rye Cover Crop and Nitrogen Fertilization Effects on Nitrate Leaching in Inbred Maize Fields. J. Environ. Qual. 29, 298 (2000).

51. Rivers, A., Mullen, C., Wallace, J. & Barbercheck, M. Cover crop-based reduced tillage system influences Carabidae (Coleoptera) activity, diversity and trophic group during transition to organic production. Renew. Agric. Food Syst. 32, 538–551 (2017).

52. Ritter, W. F. W., Scarborough, R. W. R. & Chirnside, A. E. M. Winter cover crops as a best management practice for reducing nitrogen leaching. J. Contam. Hydrol. 34, 1–15 (1998).

53. Sainju, U. M., Whitehead, W. F., Singh, B. P. & Wang, S. Tillage, cover crops, and nitrogen fertilization effects on soil nitrogen and cotton and sorghum yields. Eur. J. Agron. 25, 372–382 (2006).

54. Schipanski, M. E. et al. A framework for evaluating ecosystem services provided by cover crops in agroecosystems. Agric. Syst. 125, 12–22 (2014).

55. Schutter, M. E., Sandeno, J. M. & Dick, R. P. Seasonal, soil type, and alternative management influences on microbial communities of vegetable cropping systems. Biol. Fertil. Soils 34, 397–410 (2001).

56. Schutter, M. E. & Dick, R. P. Microbial Community Profiles and Activities among Aggregates of Winter Fallow and Cover-Cropped Soil. Soil Sci. Soc. Am. J. 66, 142 (2002).

57. Cott, H. D., Keisling, T. C., Waddle, B. A., Williams, R. W. & Frans, R. E. Effects of winter cover crops on yield of cotton and soil properties. Bull. Arkansas Agric. Exp. Stn. 924, 1–24 (1990).

58. Staver, K. W. & Brinsfield, R. B. Patterns of soil nitrate availability in corn production systems - Implications for reducing groundwater contamination. J. Soil Water Conserv. 45, 318–323 (1990).

59. Staver, K. W. & Brinsfield, R. B. Using cereal grain winter cover crops to reduce groundwater nitrate contamination in the mid-Atlantic Coastal Plain. J. Soil Water Conserv. 53, 230–240 (1998).

60. Steele, M. K., Coale, F. J. & Hill, R. L. Winter Annual Cover Crop Impacts on No-Till Soil Physical Properties and Organic Matter. Soil Sci. Soc. Am. J. 76, 2164 (2012).

61. Stipesevic, B. Effects of wheat cover crop desiccation times on soil physical properties and early growth of corn under no-till and conventional tillage systems. 9, (Purdue university, 2003).

62. Strock, J. S., Porter, P. M. & Russelle, M. P. Cover cropping to reduce nitrate loss through subsurface drainage in the Northern U.S. corn belt. J. Environ. Qual. 33, 1010–1016 (2004).

63. Teasdale, J. R. & Mohler, C. L. Light Transmittance, Soil Temperature, and Soil Moisture under Residue of Hairy Vetch and Rye. Agron. J. 85, 673 (1993).

64. Terra, J. A., Reeves, D. W., Shaw, J. N. & Raper, R. L. Impacts of landscape attributes on carbon sequestration during the transition from conventional to conservation management practices on a Coastal Plain field. J. Soil Water Conserv. 60, 438–446 (2005).

65. Sattell, R. Using Cover Crops in Oregon. Oregon State Univ. Ext. Serv. EM 8704, 1–54 (1998).

66. Veum, K. S. et al. Conservation effects on soil quality indicators in the Missouri Salt River Basin. J. Soil Water Conserv. 70, 232–246 (2015).

67. Villamil, M. . B. et al. No-Till Corn / Soybean Systems Including Winter Cover Crops : Effects on Soil Properties. Soil Sci. Soc. Am. J. 70, 1936–1944 (2006).

68. Jokela, W., Posner, J., Hedtcke, J., Balser, T. & Read, H. Midwest cropping system effects on soil properties and on a soil quality index. Agron. J. 103, 1552–1562 (2011).

69. Williams, M. M., Mortensen, D. A. & Doran, J. W. No-tillage soybean performance in cover crops for weed management in the western Corn Belt. J. Soil Water Conserv. 55, 79–84 (2000).

70. Wyland, L. J. et al. Altering surface soil dynamics with winter cover crops in a vegetable cropping system: impacts on yield, nitrate leaching, pests and management costs. Agric. Ecosyst. Environ. 59, 1–17 (1996).

71. Yoo, K. H., Touchton, J. T. & Walker, R. H. Runoff , Sediment and Nutrient Losses from Various Tillage Systems of Cotton. Soil Tillage Res. 12, 13–24 (1988).

72. Zelles, L., Bai, Q. Y., Beck, T. & Beese, F. Signature Fatty-Acids in Phospholipids and Lipopolysaccharides As Indicators of Microbial Biomass and Community Structure in Agricultural Soils. Soil Biol. Biochem. 24, 317–323 (1992).

73. Zentner, R. P. et al. Long-term assessment of management of an annual legume green manure crop for fallow replacement in the Brown soil zone. Can. J. Plant Sci. 84, 11–22 (2004).

74. Zhou, X. G. & Liu, G. Efficacy of combined use of brassica biofumigant crop and PGPR strain for managing sheath blight in rice, 2012. in Plant Disease Management Reports (2013). doi:10.1111/j.1365-3180.2010.00783.x

75. Zhu, J. C., Gantzer, C. J., Anderson, S. H., Alberts, E. E. & Beuselinck, P. R. Runoff, Soil, and Dissolved Nutrient Losses from No-Till Soybean with Winter Cover Crops. Soil Sci. Soc. Am. J. 53, 1210 (1989).

76. Angers, D. A., Samson, N. & Légère, A. Early changes in water-stable aggregation induced by rotation and tillage in a soil under barley production. Can. J. Soil Sci. 73, 51–59 (1993).

77. Beare, M. H. & Bruce, R. R. A comparison of methods for measuring water-stable aggregates - implications for determining environmental-effects on soil structure. Geoderma 56, 87–104 (1993).

78. Benjamin, J. G., Mikha, M. M. & Vigil, M. F. Organic carbon effects on soil physical and hydraulic properties in a semiarid climate. Soil Sci. Soc. Am. J. 72, 1357 (2008).

79. Drinkwater, L. E., Wagoner, P. & Sarrantonio, M. Legume-based cropping systems have reuced carbon and nitrogen losses. Nature 396, 262–265 (1998).

80. Brill, G. D. & Neal, O. R. Seasonal occurrence of runoff and erosion from a sandy soil in vegetable production. Agron. J. 42, 192–195 (1950).

81. Chu, B., Zaid, F. & Eivazi, F. Long-Term Effects of Different Cropping Systems on Selected Enzyme Activities. Commun. Soil Sci. Plant Anal. 47, 720–730 (2016).

82. Jewett, M. R. & Thelen., K. D. Winter cereal cover crop removal strategy affects spring soil nitrate levels. J. Sustain. Agric. 29, 56–67 (2007).

83. Nyakatawa, E. Tillage, cover cropping, and poultry litter effects on selected soil chemical properties. Soil Tillage Res. 58, 69–79 (2001).

84. Lupwayi Z., N., Rice A., W. & Clayton W., G. Soil microbial diversity and community structure under wheat as influenced by tillage and crop rotation. Soil Biol. Biochem. 30, 1733–1741 (1998).

85. Robb, D., Zehnder, G., Kloot, R., Bridges, W. & Park, D. Weeds , nitrogen and yield : measuring the effectiveness of an organic cover cropped vegetable no-till system. Renew. Agric. Food Syst. 1–8 (2018).

86. Anderson, S. H., Udawatta, R. P., Seobi, T. & Garrett, H. E. Soil water content and infiltration in agroforestry buffer strips. Agrofor. Syst. 75, 5–16 (2009).

87. Eckert, D. J. Chemical Attributes of Soils Subjected to No-Till Cropping with Rye Cover Crops. Soil Sci. Soc. Am. J. 55, 405–409 (1991).

88. Hermawan, B. & Bomke, A. A. Effects of winter cover crops and successive spring tillage on soil aggregation. Soil Tillage Res. 44, 109–120 (1997).

89. Kuo, S., Sainju, U. M. & Jellum., E. J. Winter Cover Crop Effects on Soil Organic Carbon and Carbohydrate in Soil. Soil Sci. Soc. Am. J. 61, 145–152 (2017).

90. Entry, J. A., Mitchell, C. C. & Backman, C. B. Influence of management practices on soil organic matter, microbial biomass and cotton yield in Alabama’s ‘Old Rotation’. Biol. Fertil. Soils 23, 353–358 (1996).

91. Sainju, U. M., Singh, B. P., Whitehead, W. F. & Wang, S. Carbon Supply and Storage in Tilled and Nontilled Soils as Influenced by Cover Crops and Nitrogen Fertilization. J. Environ. Qual. 35, 1507 (2006).

92. Sainju, U. M., Whitehead, W. F. & Singh, B. P. Cover crops and nitrogen fertilization effects on soil aggregation and carbon and nitrogen pools. Can. J. Soil Sci. 83, 155–165 (2003).

93. Sainju, U. ., Singh, B. . & Whitehead, W. . Long-term effects of tillage, cover crops, and nitrogen fertilization on organic carbon and nitrogen concentrations in sandy loam soils in Georgia, USA. Soil Tillage Res. 63, 167–179 (2002).

94. Sainju, U. M. et al. Cover crop effect on soil carbon fractions under conservation tillage cotton. Soil Tillage Res. 96, 205–218 (2007).

95. Sainju, U. M., Lenssen, A., Caesar-Thonthat, T. & Waddell, J. Dryland plant biomass and soil carbon and nitrogen fractions on transient land as influenced by tillage and crop rotation. Soil Tillage Res. 93, 452–461 (2007).

96. Utomo, M., Frye, W. W. & Blevins, R. L. Sustaining Soil Nitrogen for Corn Using Hairy Vetch Cover Crop. Agron. J. 82, 979 (1990).

97. Abdallahi, M. M. & N’Dayegamiye, A. Effets de deux incorporations d’engrais verts sur le rendement et la nutrition en azote du blé ( Triticum aestivum L.), ainsi que sur les propriétés physiques et biologiques du sol. Can. J. Soil Sci. 80, 81–89 (2000).

98. Amado, T. J. C. et al. Potential of Carbon Accumulation in No-Till Soils with Intensive Use and Cover Crops in Southern Brazil. J. Environ. Qual. 35, 1599 (2006).

99. Baumecker, M., Ellmer, F. & Köhn, W. Statischer Nährstoffmangelversuch Thyrow. Dauerfeldversuche in Brandenburg und Berlin. Beiträge für eine nachhaltige landwirtschaftliche Bodennutzung. Potsdam, Germany: Ministerium für Ländliche Entwicklung. Umwelt und Verbraucherschutz (2009).

100. Bayer, C., Mielniczuk, J., Amado, T. J. C., Mantin-Neto, L. & Fernandes, S. V. Organic matter storage in a sandy clay loamAcrisol affected by tillage and cropping systems in southern Brazil. Soil Tillage Res. 54, 101–109 (2000).

101. Constantin, J. et al. Effects of catch crops, no till and reduced nitrogen fertilization on nitrogen leaching and balance in three long-term experiments. Agric. Ecosyst. Environ. 135, 268–278 (2010).

102. Curtin, D. et al. Legume green manure as partial fallow replacement in semiarid Saskatchewan: Effect on carbon fluxes. Can. J. Soil Sci. 80, 499–505 (2000).

103. Barkusky, D. et al. Dauerfeldversuche in Brandenburg und Berlin-Beiträge fuer eine nachhaltige landwirtschaftliche Bodennutzung. Schriftenr. des Landesamtes fuer Verbraucherschutz, Landwirtschaft und Flurneuordnung Abteilung Landwirtschaft und Gartenbau, R. Landwirtschaft 10, 4 (2009).

104. Mazzoncini, M., Sapkota, T. B., Bàrberi, P., Antichi, D. & Risaliti, R. Long-term effect of tillage, nitrogen fertilization and cover crops on soil organic carbon and total nitrogen content. Soil Tillage Res. 114, 165–174 (2011).

105. Metay, A. et al. Storage and forms of organic carbon in a no-tillage under cover crops system on clayey Oxisol in dryland rice production (Cerrados, Brazil). Soil Tillage Res. 94, 122–132 (2007).

106. Nascente, A. S., Crusciol, C. A. C., Cobucci, T. & Velini, E. D. Cover crop termination timing on rice crop production in a no-till system. Crop Sci. 53, 2659–2669 (2013).

107. Sadat-dastegheibi, B. Untersuchungen zur stoffdynamik in ackerboden in abhangigkeit v... (1974).

108. Witter, E. Soil C balance in a long-term field experiment in relation to the size of the microbial biomass. Biol. Fertil. Soils 23, 33–37 (1996).

109. Thomsen, I. K., Thomsen, I. K. & Christensen, B. T. Yields of wheat and soil carbon and nitrogen contents following long-term incorporation of barley straw and ryegrass catch crops. Soil Use Manag. 20, 432–438 (2004).

110. Wilson, G. F., Lal, R. & Okigbo, B. N. Effects of cover crops on soil structure and on yield of subsequent arable crops grown under strip tillage on an eroded Alfisol. Soil Tillage Res. 2, 233–250 (1982).

111. Alvarez, R., Steinbach, H. S. & De Paepe, J. L. Cover crop effects on soils and subsequent crops in the pampas: A meta-analysis. Soil Tillage Res. 170, 53–65 (2017).

112. Abdollahi, L. & Munkholm, L. J. Tillage System and Cover Crop Effects on Soil Quality:I. Chemical, Mechanical, and Biological Properties. Soil Sci. Soc. Am. J. 78, 262 (2014).

113. Abdollahi, L., Munkholm, L. J. & Garbout, A. Tillage System and Cover Crop Effects on Soil Quality: II. Pore Characteristics. Soil Sci. Soc. Am. J. 78, 271 (2014).

114. Ward, P. R., Flower, K. C., Cordingley, N., Weeks, C. & Micin, S. F. Soil water balance with cover crops and conservation agriculture in a Mediterranean climate. F. Crop. Res. 132, 33–39 (2012).

115. Whish, J. P. M., Price, L. & Castor, P. A. Do spring cover crops rob water and so reduce wheat yields in the northern grain zone of eastern Australia? Crop Pasture Sci. 60, 517–525 (2009).

116. Abunyewa, A. A. & Karbo, K. N. Improved fallow with pigeon pea for soil fertility improvement and to increase maize production in a smallholder crop-livestock farming system in the subhumid zone of Ghana. L. Degrad. Dev. 16, 447–454 (2005).

117. Agyare, W. A., Kombiok, J. M., Karbo, N. & Larbi, A. Management of pigeon pea in short fallows for crop-livestock production systems in the Guinea savanna zone of northern Ghana. Agrofor. Syst. 54, 197–202 (2002).

118. Akinnifesi, F. K., Makumba, W. & Kwesiga, F. R. Sustainable maize production using gliricidia/maize intercropping in southern Malawi. Exp. Agric. 42, 441–457 (2006).

119. Akinnifesi, F. K., Makumba, W., Sileshi, G., Ajayi, O. C. & Mweta, D. Synergistic effect of inorganic N and P fertilizers and organic inputs from Gliricidia sepium on productivity of intercropped maize in Southern Malawi. Plant Soil 294, 203–217 (2007).

120. Böhringer, A. & Leihner., D. E. Pigeonpea in simultaneous fallow-cropping systems in the subhumid forest-savanna mosaic zone of West Africa. Exp. Agric. 33, 301-312. (1997).

121. Carsky, R. J., Oyewole, B. & Tian, G. Integrated soil management for the savanna zone of W. Africa: Legume rotation and fertilizer N. Nutr. Cycl. Agroecosystems 55, 95–105 (1999).

122. Chintu, R. et al. Propagation and management of gliricidia sepium planted fallows in sub-humid eastern Zambia. Exp. Agric. 40, 341–352 (2004).

123. Chirwa, T. S., Mafongoya, P. L. & Chintu, R. Mixed planted-fallows using coppicing and non-coppicing tree species for degraded Acrisols in eastern Zambia. Agrofor. Syst. 59, 243–251 (2003).

124. Chirwa, T. S., Mafongoya, P. L., Mbewe, D. N. M. & Chishala, B. H. Changes in soil properties and their effects on maize productivity following Sesbania sesban and Cajanus cajan improved fallow systems in eastern Zambia. Biol. Fertil. Soils 40, 20–27 (2004).

125. Cooper, P. J. M., Leakey, R. R., Rao, M. R. & Reynolds, L. Agroforestry and the mitigation of land degradation in the humid and sub-humid tropics of Africa. Exp. Agric. 32, 235–290 (1996).

126. Banda, A. Z., Maghembe, J. A., Ngugi, D. N. & Chome, V. A. Effect of intercropping maize and closely spaced Leucaena hedgerows on soil conservation and maize yield on a steep slope at Ntcheu, Malawi. Agrofor. Syst. 27, 17–22 (1994).

127. Drechsel, P., Steiner, K. G. & Hagedorn, F. A review on the potential of improved fallows and green manure in Rwanda. Agrofor. Syst. 33, 109–136 (1996).

128. Esilaba, A. O. et al. On farm testing of integrated nutrient management strategies in eastern Uganda. Agric. Syst. 86, 144–165 (2005).

129. Fischler, M. & Wortmann, C. S. Green manures for maize – bean systems in eastern Uganda : Agronomic performance and farmers ’ perceptions. Agrofor. Syst. 47, 123–138 (1999).

130. Fischler, M., Wortmann, C. S. & Feil, B. Crotalaria (C-ochroleuca G Don) as a green manure in maize-bean cropping systems in Uganda. F. Crop. Res. 61, 97–107 (1999).

131. Fofana, B. et al. Using mucuna and P fertilizer to increase maize grain yield and N fertilizer use efficiency in the coastal savanna of Togo. Nutr. Cycl. Agroecosystems 68, 213–222 (2004).

132. Franke, A. C., Schulz, S., Oyewole, B. D. & Bako, S. Incorporating short-season legumes and green manure crops into maize-based systems in the moist Guinea savanna of West Africa. Exp. Agric. 40, 463–479 (2004).

133. Gacheru, E. & Rao, M. R. The potential of planted shrub fallows to combat Striga infestation on maize. Int. J. Pest Manag. 51, 91–100 (2005).

134. Gichuru, M. P. Residual Effects of Natural Bush, Cajanus-Cajan and Tephrosia-Candida on the Productivity of an Acid Soil in Southeastern Nigeria. Plant Soil 134, 31–36 (1991).

135. Harawa, R., Lehmann, J., Akinnifesi, F., Fernandes, E. & Kanyama-Phiri, G. Nitrogen dynamics in maize-based agroforestry systems as affected by landscape position in southern Malawi. Nutr. Cycl. Agroecosystems 75, 271–284 (2006).

136. Ikpe, F. N., Owoeye, L. G. & Gichuru, M. P. Nutrient recycling potential of Tephrosia candida in cropping systems of southeastern Nigeria. Nutr. Cycl. Agroecosystems 67, 129–136 (2003).

137. Egbe, E. A., Ladipo, D. O., Nwoboshi, L. C. & Swift, M. J. Potentials of Millettia thonningii and Pterocarpus santalinoides for alley cropping in humid lowlands of West Africa. Agrofor. Syst. 40, 309–321 (1998).

138. Jama, B., Swinkels, R. A. & Buresh., R. J. Agronomic and economic evaluation of organic and inorganic sources of phosphorus in western Kenya. Agron. J. 89, 597–604 (1997).

139. Jama, B., Buresh, R. J. & Place, F. M. Sesbania tree fallows on phosphorus-deficient sites: Maize yield and financial benefit. Agron. J. 90, 717–726 (1998).

140. Jeranyama, P., Hesterman, O. B., Waddington, S. R. & Harwood, R. R. Relay-intercropping of sunnhemp and cowpea into a smallholder maize system in Zimbabwe. Agron. J. 92, 239–244 (2000).

141. Kaho, F., Yemefack, M., Nguimgo, B. A. K. & Zonkeng, C. G. The Effect of Short Rotation Desmodium distortum Planted Fallow on the Productivity of Uitisols in Centre Cameroon. Tropicultura 22, 49–55 (2004).

142. Kaizzi, C. K., Ssali, H. & Vlek, P. L. G. Differential use and benefits of Velvet bean (Mucuna pruriens var. utilis) and N fertilizers in maize production in contrasting agro-ecological zones of E. Uganda. Agric. Syst. 88, 44–60 (2006).

143. Kamanga, B. C. G., G. Y. K.-P. & Minae, S. Intercropping perennial legumes for green manure additions to maize in southern Malawi. African Crop Sci. J. 7, 355–363 (1999).

144. König, D. The potential of agroforestry methods for erosion control in Rwanda. Soil Technol. 5, 167–176 (1992).

145. Kwesiga, F. & Coe, R. The effect of short rotation Sesbania sesban planted fallows on maize yield. For. Ecol. Manage. 64, 199–208 (1994).

146. Kwesiga, F. R., Franzel, S., Place, F., Phiri, D. & Simwanza, C. P. Sesbania sesban improved fallows in eastern Zambia: Their inception, development and farmer enthusiasm. Agrofor. Syst. 47, 49–66 (1999).

147. Lal, R. Agroforestry Systems and Soil Surface Management of a Tropical Alfisol .II: Water Runoff, Soil-Erosion, and Nutrient Loss. Agroforest Syst 8, 97–111 (1989).

148. Maccoll, D. Studies on maize (Zea mays) at Bunda, Malawi. III. Yield in rotations with pasture legumes. Exp. Agric. 26, 263–271 (1990).

149. Mafongoya, P. & Dzowela, B. Biomass production of tree fallows and their residual effect on maize in Zimbabwe. Agrofor. Syst. 47, 139–151 (1999).

150. Mafongoya, P. L., Bationo, A., Kihara, J. & Waswa, B. S. Appropriate technologies to replenish soil fertility in southern Africa. Nutr. Cycl. Agroecosystems 76, 137–151 (2006).

151. Maroko, J. B., Buresh, R. J. & Smithson, P. C. Soil Phosphorus Fractions in Unfertilized Fallow-Maize Systems on Two Tropical Soils. Soil Sci. Soc. Am. J. 63, 320 (1999).

152. Morse, S. & McNamara, N. Factors affecting the adoption of leguminous cover crops in Nigeria and a comparison with the adoption of new crop varieties. Exp. Agric. 39, 81–97 (2003).

153. Muleba, N. Effects of cowpea, crotalaria and sorghum crops and phosphorus fertilizers on maize productivity in semi-arid West Africa. J. Agric. Sci. 132, 61–70 (1999).

154. Niang, A. I., Amadalo, B. A., De Wolf, J. & Gathumbi, S. M. Species screening for short-term planted fallows in the highlands of western Kenya. Agrofor. Syst. 56, 145–154 (2002).

155. Njunie, M. N. & Wagger, M. G. Use of Herbaceous Legumes for Improving Soil Fertility and Crop Yield in Maize Cassava Cropping Systems. East African Agric. For. J. 69, 49–61 (2003).

156. Nyadzi, G. I. et al. Rotational woodlot technology in northwestern Tanzania: Tree species and crop performance. Agrofor. Syst. 59, 253–263 (2003).

157. Onim, J. F. M., Mathuva, M., Otieno, K. & Fitzhugh, H. A. Soil fertility changes and response of maize and beans to green manures of leucaena, sesbania and pigeonpea. Agrofor. Syst. 12, 197–215 (1990).

158. Phiri, a D. K. & Snapp, S. Maize and sesbania production in relay cropping at three landscape positions in Malawi. Agrofor. Syst. 47, 153–162 (1999).

159. Phiri, R. H., Kanyama-Phiri, G. Y. & Snapp, S. Soil nitrate dynamics in relation to nitrogen source and landscape position in Malawi. Agrofor. Syst. 47, 253–262 (1999).

160. Phiri, E., Verplancke, H., Kwesiga, F. & Mafongoya, P. Water balance and maize yield following improved sesbania fallow in eastern Zambia. Agrofor. Syst. 59, 197–205 (2003).

161. Rao, M. R. et al. Duration of sesbania fallow effect for nitrogen requirement of maize in planted fallow-maize rotation in Western Kenya. Exp. Agric. 38, 223–236 (2002).

162. Myaka, F. M. et al. Yields and accumulations of N and P in farmer-managed intercrops of maize-pigeonpea in semi-arid Africa. Plant Soil 285, 207–220 (2006).

163. Sakala, W. D., Kumwenda, J. D. T. & Saka, A. R. The potential of green manure to increase soil fertility and maize yield.". Biol. Agric. Hortic. 2, 121–130 (2003).

164. Shirima, D. S., Otsyina, R., Mwageni, W. P. & Bridge, J. Effect of natural and Sesbania fallows and crop rotations on the incidence of root-knot nematodes and tobacco production in Tabora, Tanzania. Int. J. Nematol. 10, 49–54 (2000).

165. Sileshi, G. & Mafongoya, P. L. Effect of rotational fallows on abundance of soil insects and weeds in maize crops in eastern Zambia. Appl. Soil Ecol. 23, 211–222 (2003).

166. Sileshi, G. & Mafongoya, P. L. Long-term effects of improved legume fallows on soil invertebrate macrofauna and maize yield in eastern Zambia. Agric. Ecosyst. Environ. 115, 69–78 (2006).

167. Sileshi, G. & Mafongoya, P. L. Variation in macrofaunal communities under contrasting land use systems in eastern Zambia. Appl. Soil Ecol. 33, 49–60 (2006).

168. Sileshi, G., Mafongoya, P. L., Kwesiga, F. & Nkunika, P. Termite damage to maize grown in agroforestry systems, traditional fallows and monoculture on nitrogen-limited soils in eastern Zambia. Agric. For. Entomol. 7, 61–69 (2005).

169. Thor Smestad, B., Tiessen, H. & Buresh, R. J. Short fallows of Tithonia diversifolia and Crotalaria grahamiana for soil fertility improvement in western Kenya. Agrofor. Syst. 55, 181–194 (2002).

170. Sogbedji, J. M., van Es, H. & Agbeko, K. L. Cover Cropping and Nutrient Management Strategies for Maize Production in Western Africa. Agron. J. 98, 883 (2006).

171. Tian, G., Kolawole, G. O., Kang, B. T. & Kirchhof, G. Nitrogen fertilizer replacement indexes of legume cover crops in the derived savanna of West Africa. Plant Soil 224, 287–296 (2000).

172. Tian, G., Kang, B. T., Kolawole, G. O., Idinoba, P. & Salako, F. K. Long-term effects of fallow systems and lengths on crop production and soil fertility maintenance in West Africa. Nutr. Cycl. Agroecosystems 71, 139–150 (2005).

173. Torquebiau, E. F. & Kwesiga, F. Root development in a Sesbania sesban fallow-maize system in Eastern Zambia. Agrofor. Syst. 34, 193–211 (1996).

174. Chen, L. L., You, M. S. & Chen, S. B. Effects of cover crops on spider communities in tea plantations. Biol. Control 59, 326–335 (2011).

175. Cui, H. et al. The combined effects of cover crops and symbiotic microbes on phosphatase gene and organic phosphorus hydrolysis in subtropical orchard soils. Soil Biol. Biochem. 82, 119–126 (2015).

176. Du, S., Bai, G. & Yu, J. Soil properties and apricot growth under intercropping and mulching with erect milk vetch in the loess hilly-gully region. Plant Soil 390, 431–442 (2015).

177. Gao, D., Zhou, X., Duan, Y., Fu, X. & Wu, F. Wheat cover crop promoted cucumber seedling growth through regulating soil nutrient resources or soil microbial communities? Plant Soil 418, 459–475 (2017).

178. Habbib, H. et al. Investigating the Combined Effect of Tillage, Nitrogen Fertilization and Cover Crops on Nitrogen Use Efficiency in Winter Wheat. Agronomy 7, 66 (2017).

179. Haque, M. M., Kim, S. Y., Pramanik, P., Kim, G. Y. & Kim, P. J. Optimum application level of winter cover crop biomass as green manure under considering methane emission and rice productivity in paddy soil. Biol. Fertil. Soils 49, 487–493 (2013).

180. Isik, D., Kaya, E., Ngouajio, M. & Mennan, H. Weed suppression in organic pepper (Capsicum annuum L.) with winter cover crops. Crop Prot. 28, 356–363 (2009).

181. Kim, S. Y., Lee, C. H., Gutierrez, J. & Kim, P. J. Contribution of winter cover crop amendments on global warming potential in rice paddy soil during cultivation. Plant Soil 366, 273–286 (2013).

182. Liu, J. N. et al. Winter cover crops alter methanotrophs community structure in a double-rice paddy soil. J. Integr. Agric. 15, 553–565 (2016).

183. Min, J., Shi, W., Xing, G., Zhang, H. & Zhu, Z. Effects of a catch crop and reduced nitrogen fertilization on nitrogen leaching in greenhouse vegetable production systems. Nutr. Cycl. Agroecosystems 91, 31–39 (2011).

184. Tang, H. M. et al. Effects of winter cover crops straws incorporation on CH4 and N2O emission from double-cropping paddy fields in southern China. PLoS One 9, (2014).

185. Hai-Ming, T. et al. Effects of Winter Cover Crops Residue Returning on Soil Enzyme Activities and Soil Microbial Community in Double-Cropping Rice Fields. PLoS One 9, e100443 (2014).

186. Tang, H. et al. Effects of winter covering crop residue incorporation on CH4and N2O emission from double-cropped paddy fields in southern China. Environ. Sci. Pollut. Res. 22, 12689–12698 (2015).

187. Tian, Y., Zhang, X., Liu, J. & Gao, L. Effects of summer cover crop and residue management on cucumber growth in intensive Chinese production systems: Soil nutrients, microbial properties and nematodes. Plant Soil 339, 299–315 (2011).

188. Zheng, W. et al. Improving yield and water use efficiency of apple trees through intercrop-mulch of crown vetch (Coronilla varia L.) combined with different fertilizer treatments in the Loess Plateau. Spanish J. Agric. Res. 14, (2016).

189. Xie, Z., Shah, F., Tu, S., Xu, C. & Cao, W. Chinese milk vetch as green manure mitigates nitrous oxide emission from monocropped rice system in South China. PLoS One 11, 1–16 (2016).

190. Yu, Y., Xue, L. & Yang, L. Winter legumes in rice crop rotations reduces nitrogen loss, and improves rice yield and soil nitrogen supply. Agron. Sustain. Dev. 34, 633–640 (2014).

191. Yuan, H. M., Blackwell, M., Rahn, C. & Chen, Q. Fertilization and Catch Crop Strategies for Improving Tomato Production in North China. Pedosphere 25, 364–371 (2015).

192. Dabin, Z. et al. Responses of winter wheat production to green manure and nitrogen fertilizer on the loess plateau. Agron. J. 107, 361–374 (2015).

193. Dabin, Z. et al. Contribution of green manure legumes to nitrogen dynamics in traditional winter wheat cropping system in the Loess Plateau of China. Eur. J. Agron. 72, 47–55 (2016).

194. Zhang, D. et al. Soil Water Balance and Water Use Efficiency of Dryland Wheat in Different Precipitation Years in Response to Green Manure Approach. Sci. Rep. 6, 1–12 (2016).

195. Zhu, B. et al. Performance of two winter cover crops and their impacts on soil properties and two subsequent rice crops in Dongting Lake Plain, Hunan, China. Soil Tillage Res. 124, 95–101 (2012).

196. Zhu, B. et al. Non-leguminous winter cover crop and nitrogen rate in relation to double rice grain yield and nitrogen uptake in Dongting Lake Plain, Hunan Province, China. J. Integr. Agric. 15, 2507–2514 (2016).

197. Clark, K. M. et al. Crop yield and soil organic carbon in conventional and no-till organic systems on a claypan soil. Agron. J. 109, 588–599 (2017).

198. García-Orenes, F. et al. Organic Fertilization in Traditional Mediterranean Grapevine Orchards Mediates Changes in Soil Microbial Community Structure and Enhances Soil Fertility. L. Degrad. Dev. 27, 1622–1628 (2016).

199. Marques, M. J., García-Muñoz, S., Muñoz-Organero, G. & Bienes, R. Soil conservation beneath grass cover in hillside vineyards under mediterranean climatic conditions (MADRID, SPAIN). L. Degrad. Dev. 21, 122–131 (2010).

200. Montanaro, G., Celano, G., Dichio, B. & Xiloyannis, C. Effects of soil‐- protecting agricultural practices on soil organic carbon and productivity in fruit tree orchards. L. Degrad. Dev. 21, 132–138 (2010).

201. Moore, E. B., Wiedenhoeft, M. H., Kaspar, T. C. & Cambardella, C. A. Rye Cover Crop Effects on Soil Quality in No-Till Corn Silage–Soybean Cropping Systems. Soil Sci. Soc. Am. J. 78, 968 (2014).

202. Olson, K., Ebelhar, S. A. & Lang, J. M. Long-Term Effects of Cover Crops on Crop Yields, Soil Organic Carbon Stocks and Sequestration. Open J. Soil Sci. 04, 284–292 (2014).

203. Piccoli, I., Camarotto, C., Lazzaro, B., Furlan, L. & Morari, F. Conservation Agriculture Had a Poor Impact on the Soil Porosity of Veneto Low-lying Plain Silty Soils after a 5-year Transition Period. L. Degrad. Dev. 28, 2039–2050 (2017).

204. Sporton, D. CULTIVATING SUCCESS IN UGANDA: KIGEZI FARM- ERSANDCOLONIAL POLICIES, Carswell Grace. L. Degrad. Dev. 20, 587–588 (2009).

205. Repullo-Ruibérriz de Torres, M. A. et al. Efficiency of four different seeded plants and native vegetation as cover crops in the control of soil and carbon losses by water erosion in olive orchards. L. Degrad. Dev. 29, 2278–2290 (2018).

206. Wiesmeier, M. et al. Rebuilding soil carbon in degraded steppe soils of Eastern Europe: The importance of windbreaks and improved cropland management. L. Degrad. Dev. 29, 875–883 (2018).

207. Andrews, S. S. et al. On-Farm Assessment of Soil Quality in California ’ s Central Valley. Agron. J. 94, 12–23 (2002).

208. Brown, S. A., Cook, H. F. & Lee, H. C. Topsoil characteristics from a paired farm survey of organic versus conventional farming in southern England. Biol. Agric. Hortic. 18, 37–54 (2000).

209. Benitez, E., Nogales, R., Campos, M. & Ruano, F. Biochemical variability of olive-orchard soils under different management systems. Appl. Soil Ecol. 32, 221–231 (2006).

210. Blaise, D. Yield, boll distribution and fibre quality of hybrid cotton (Gossypium hirsutum L.) as influenced by organic and modern methods of cultivation. J. Agron. Crop Sci. 192, 248–256 (2006).

211. Blakemore, R. J. Ecology of earthworms under the ‘Haughley Experiment’of organic and conventional management regimes. Biol. Agric. Hortic. 18, 41–159 (2000).

212. Campos-Herrera, R., Piedra-Buena, A., Escuer, M., Montalbán, B. & Gutiérrez, C. Effect of seasonality and agricultural practices on occurrence of entomopathogenic nematodes and soil characteristics in La Rioja (Northern Spain). Pedobiologia (Jena). 53, 253–258 (2010).

213. Canali, S. et al. Effect of different management strategies on soil quality of citrus orchards in Southern Italy. Soil Use Manag. 25, 34–42 (2009).

214. Diez, T. et al. Vergleichende Bodenuntersuchungen von konventionell und alternativ bewirtschafteten Betriebsschlägen. Bayer. Landwirtsch. Jahrb. 63, 979–1019 (1986).

215. Chirinda, N., Olesen, J. E., Porter, J. R. & Schjønning, P. Soil properties, crop production and greenhouse gas emissions from organic and inorganic fertilizer-based arable cropping systems. Agric. Ecosyst. Environ. 139, 584–594 (2010).

216. Ciavatta, C., Gioacchini, P. & Montecchio, D. Can organic farming contribute to carbon sequestration? A survey in a pear orchard in Emilia-Romagna region, Italy. in 16th IFOAM Organic World Congress, Modena, Italy 16–20 (2008).

217. Clark, M. S., Horwarth, W. R., Shennan, C. & Scow, K. M. Changes in soil chemical properties resulting fron organic and low-input farming practices. Agron. Journal. 90., 662-671. (1998).

218. Delate, K. & Cambardella, C. A. Agroecosystem performance during transition to certified organic grain production. Agron. J. 96, 1288–1298 (2004).

219. Deria, A. M., Bell, R. W. & O’hara, G. W. Organic Wheat Production and Soil Nutrient Status in a Mediterranean Climatic Zone. J. Sustain. Agric. 21, 21–47 (2003).

220. Derrick, J. W. & Dumaresq, D. C. Soil properties under organic and conventional management in southern New South Wales. Soil Res. 37, 1047–1056 (1999).

221. Diez, T., Bihler, E. & Krauss, M. Auswirkungen abgestufter Intensitäten im Pflanzenbau auf Lebensgemeinschaften des Ackers, Bodenfruchtbarkeit und Ertrag. IV. Auswirkungen abgestufter Pflanzenbauintensitäten auf Bodenkennwerte und Nährstoffbilanz. Bayer. Landw. Jahrb 68, 354–361 (1991).

222. Dilly, O., Winter, K., Lang, A. & Munch, J. C. Energetic eco-physiology of the soil microbiota in two landscapes of southern and northern Germany. J. Plant Nutr. Soil Sci. 164, 407–413 (2001).

223. Droogers, P. & Bouma, J. Biodynamic vs. Conventional Farming Effects on Soil Structure Expressed by Simulated Potential Productivity. Soil Sci. Soc. Am. J. 60, 1552 (1996).

224. Efthimiadou, E., Papatheodorou, E. M., Monokrousos, N. & Stamou, G. P. Changes of soil chemical, microbiological, and enzymatic variables in relation to management regime and the duration of organic farming in Phaseolus vulgaris. J. Biol. Res. 14, 151–159 (2010).

225. Eltun, R., Kors‘th, A. & Nordheim, O. A comparison of environmental, soil fertility, yield, and economical effects in six cropping sysrtems based on an 8- years experiment in Norway. Agric. Ecosyst. Environ. 90, 155–168 (2002).

226. Eyhorn, F., Ramakrishnan, M. & Maeder, P. The viability of cotton-based organic farming systems in India. Int. J. Agric. Sustain. 5, 25–38 (2007).

227. Fraser, D. G., Doran, J. W., Sahs, W. W. & Lesoing, G. W. Soil Microbial Populations and Activities under Conventional and Organic Management. J. Environ. Qual. 17, 585 (1988).

228. Friedel, J. K. The effect of farming system on labile fractions of organic matter in Calcari-Epileptic Regosols. J. Plant Nutr. Soil Sci. 163, 41–45 (2000).

229. García-Ruiz, R. et al. Soil enzymes, nematode community and selected physico-chemical properties as soil quality indicators in organic and conventional olive oil farming: Influence of seasonality and site features. Appl. Soil Ecol. 41, 305–314 (2009).

230. Gardner, J. C. & Clancy, S. A. Impact of Farming Practices on Soil Quality in North Dakota. Methods Assess. soil Qual. methodsforasses 337–343 (1996). doi:10.1017/S000748530002229X

231. Ge, T. et al. Chemical properties, microbial biomass, and activity differ between soils of organic and conventional horticultural systems under greenhouse and open field management: A case study. J. Soils Sediments 11, 25–36 (2011).

232. Gerhardt, R. A. A Comparative Analysis of the Effects of Organic and Conventional Farming Systems on Soil Structure. Biol. Agric. Hortic. 14, 139–157 (1997).

233. Glover, J. D., Reganold, J. P. & Andrews, P. K. Systematic method for rating soil quality of conventional, organic,\rand integrated apple orchards in Washington State. Agric. Ecosyst. Environ. 80, 29–45 (2000).

234. Gosling, P. & Shepherd, M. Long-term changes in soil fertility in organic arable farming systems in England, with particular reference to phosphorus and potassium. Agric. Ecosyst. Environ. 105, 425–432 (2005).

235. Grandy, A. S. & Robertson, G. P. Land-use intensity effects on soil organic carbon accumulation rates and mechanisms. Ecosystems 10, 58–73 (2007).

236. Granstedt, A. & Kjellenberg, L. Organic and biodynamic cultivation - a possible way of increasing humus capital, improving soil fertility and providing a significant carbon sink in Nordic conditions. in 16th IFOAM Organic World Congress, Modena, Italy (2008).

237. Haggar, J. et al. Coffee agroecosystem performance under full sun, shade,conventional and organic management regimes in Central America. Agrofor. Syst. 82, 285–301 (2011).

238. Heitkamp, F., Raupp, J. & Ludwig, B. Impact of fertilizer type and rate on carbon and nitrogen pools in a sandy Cambisol. Plant Soil 319, 259–275 (2009).

239. Hepperly, P. R., Jr, D. D. & Seidel, R. The Rodale Institute Farming Systems Trial 1981 to 2005: long-term analysis of organic and conventional maize and soybean cropping systems. Long-term F. Exp. Org. farming 15–31 (2006). doi:10.1017/S000748530002229X

240. Herencia, J. F., García-Galavís, P. A. & Maqueda, C. Long-Term Effect of Organic and Mineral Fertilization on Soil Physical Properties Under Greenhouse and Outdoor Management Practices. Pedosphere 21, 443–453 (2011).

241. Kahle, P., Baum, C. & Borchwardt, M. Auswirkungen mehrjähriger ökologischer Bewirtschaftung auf ausgewählte Bodeneigenschaften, dargestellt am Beispiel des Versuchsfeldes Gülzow. Mitteilungen der Landesforschungsanstalt für Landwirtschaft und Fischerei Mecklenburg-Vorpommern 33, 21–22 (2004).

242. Kirchman, H., Bergstrom, L., Katterer, T., Mattsson, L., Gesslein, S. Comparison of Long-Term Organic and Conventional Crop-Livestock ... Agron. J. 99, 960 (2007).

243. Kong, A. Y. Y., Six, J., Bryant, D. C., Denison, R. F. & van Kessel, C. The Relationship between Carbon Input, Aggregation, and Soil Organic Carbon Stabilization in Sustainable Cropping Systems. Soil Sci. Soc. Am. J. 69, 1078 (2005).

244. Kramer, S. B., Reganold, J. P., Glover, J. D., Bohannan, B. J. M. & Mooney, H. A. Reduced nitrate leaching and enhanced denitrifier activity and efficiency in organically fertilized soils. Proc. Natl. Acad. Sci. 103, 4522–4527 (2006).

245. Leifeld, J., Reiser, R. & Oberholzer, H. R. Consequences of Conventional versus Organic farming on Soil Carbon: Results from a 27-Year Field Experiment. Agron. J. 101, 1204–1218 (2009).

246. Leite, L. F. et al. Soil organic carbon and biological indicators in an Acrisol under tillage systems and organic management in north-eastern Brazil. Soil Res. 48, 258–265 (2010).

247. Liebig, M. a & Doran, J. W. Impact of organic production practices on soil quality indicators. J. Environ. Qual. 28, 1601–1609 (1999).

248. Lytton‐Hitchins, J. A., Koppi, A. J. & McBratney., A. B. The soil condition of adjacent bio‐dynamic and conventionally managed dairy pastures in Victoria, Australia. Soil Use Manag. 10, 79–87 (1994).

249. Marinari, S., Liburdi, K., Fliessbach, A. & Kalbitz, K. Effects of organic management on water-extractable organic matter and C mineralization in European arable soils. Soil Tillage Res. 106, 211–217 (2010).

250. Marinari, S., Lagomarsino, A., Moscatelli, M. C., Di Tizio, A. & Campiglia, E. Soil carbon and nitrogen mineralization kinetics in organic and conventional three-year cropping systems. Soil Tillage Res. 109, 161–168 (2010).

251. Mazzoncini, M. et al. Comparison of organic and conventional stockless arable systems: A multidisciplinary approach to soil quality evaluation. Appl. Soil Ecol. 44, 124–132 (2010).

252. Melero, S., Porras, J. C. R., Herencia, J. F. & Madejon, E. Chemical and biochemical properties in a silty loam soil under conventional and organic management. Soil Tillage Res. 90, 162–170 (2006).

253. Moeskops, B. et al. Soil microbial communities and activities under intensive organic and conventional vegetable farming in West Java, Indonesia. Appl. Soil Ecol. 45, 112–120 (2010).

254. Mulia, D. J., Huyck, L. M. & Reganold, J. P. Temporal variation in aggregate stability on conventional and alternative farms. Soil Sci. Soc. Am. J. 56, 1620–1624 (1992).

255. Murata, T. & Goh, K. M. Effects of cropping systems on soil organic matter in a pair of conventional and biodynamic mixed cropping farms in Canterbury, New Zealand. Biol. Fertil. Soils 25, 372–381 (1997).

256. Monokrousos, N., Papatheodorou, E. M., Diamantopoulos, J. D. & Stamou, G. P. Soil quality variables in organically and conventionally cultivated field sites. Soil Biol. Biochem. 38, 1282–1289 (2006).

257. Nguyen, M. L. & Haynes, R. J. Energy and labour efficiency for three pairs of conventional and alternative cropping (pasture arable) farms in Canterbury, New Zealand. Agric. Ecosyst. Environ. 52, 163–172 (1995).

258. Beste, A. & Leithold, G. Extended Spadediagnosis-an applicable field method for the evaluation of some ecologically significant soil-function-parameters in science and agricultural consulting practice. in In IFOAM 2000: the world grows organic. Proceedings 13th International IFOAM (2000). doi:10.1017/S000748530002229X

259. Okur, N., Altindişli, A., Çengel, M., Göçmez, S. & Kayikçioǧlu, H. H. Organik ve konvansiyonel tari{dotless}m alti{dotless}ndaki baǧ topraklari{dotless}nda mikrobiyal biyokütle ve enzim aktivitesi. Turkish J. Agric. For. 33, 413–423 (2009).

260. Pardo, G., Cavero, J., Aibar, J. & Zaragoza, C. Nutrient evolution in soil and cereal yield under different fertilization type in dryland. Nutr. Cycl. Agroecosystems 84, 267–279 (2009).

261. Petersen, S. O., Debosz, K., Schjønning, P., Christensen, B. T. & Elmholt, S. Phospholipid fatty acid profiles and C availability in wet-stable macro-aggregates from conventionally and organically farmed soils. Geoderma 78, 181–196 (1997).

262. Phillips, R. L. Organic Agriculture and Nitrous Oxide Emissions at Sub-Zero Soil Temperatures. J. Environ. Qual. 36, 23 (2007).

263. Probst, B., Schüler, C. & Joergensen, R. G. Vineyard soils under organic and conventional management - Microbial biomass and activity indices and their relation to soil chemical properties. Biol. Fertil. Soils 44, 443–450 (2008).

264. Pulleman, M., Jongmans, A., Marinissen, J. & Bouma, J. Effects of organic versus conventional arable farming on soil structure and organic matter dynamics in a marine loam in the Netherlands. Soil Use Manag. 19, 157–165 (2003).

265. Purin, S., Filho, O. K. & Stürmer, S. L. Mycorrhizae activity and diversity in conventional and organic apple orchards from Brazil. Soil Biol. Biochem. 38, 1831–1839 (2006).

266. Qin, Y., Liu, S., Guo, Y., Liu, Q. & Zou, J. Methane and nitrous oxide emissions from organic and conventional rice cropping systems in Southeast China. Biol. Fertil. Soils 46, 825–834 (2010).

267. Rasul, G. & Thapa, G. B. Sustainability of ecological and conventional agricultural systems in Bangladesh: An assessment based on environmental, economic and social perspectives. Agric. Syst. 79, 327–351 (2004).

268. Reganold, J. P., Palmer, A. S., Lockhart, J. C. & Macgregor, A. N. Soil Quality and Financial Performance of Biodynamic and Conventional Farms in New Zealand. Science (80-. ). 260, 344-349. (1993).

269. Reganold, J. P. et al. Fruit and soil quality of organic and conventional strawberry agroecosystems. PLoS One 5, 1–14 (2010).

270. Romanyà, J. & Rovira, P. Organic and inorganic P reserves in rain-fed and irrigated calcareous soils under long-term organic and conventional agriculture. Geoderma 151, 378–386 (2009).

271. Rühling, I., Ruser, R., Kölbl, A., Priesack, E. & Gutser, R. Kohlenstoff und Stickstoff in Agrarökosystemen. (2005).

272. Schjønning, P., Elmholt, S., Munkholm, L. J. & Debosz, K. Soil quality aspects of humid sandy loams as inﬂuenced by organic and conventional long-term management. Agric. Ecosyst. Environ. 88, 195–214 (2002).

273. Sehy, U. N2O-Freisetzungen landwirtschaftlich genutzter Böden unter dem Einfluss von Bewirtschaftungs-und Standortfaktoren. (2003).

274. Snapp, S. S., Gentry, L. E. & Harwood, R. Management intensity - not biodiversity - the driver of ecosystem services in a long-term row crop experiment. Agric. Ecosyst. Environ. 138, 242–248 (2010).

275. Teasdale, J. R., Coffman, C. B. & Mangum, R. W. Potential long-term benefits of no-tillage and organic cropping systems for grain production and soil improvement. Agron. J. 99, 1297–1305 (2007).

276. Van Diepeningen, A. D., De Vos, O. J., Korthals, G. W. & Van Bruggen, A. H. C. Effects of organic versus conventional management on chemical and biological parameters in agricultural soils. Appl. Soil Ecol. 31, 120–135 (2006).

277. Vavoulidou, E., Coors, A., Dózsa-Farkas, K. & Römbke, J. Influence of farming practice, crop type and soil properties on the abundance of Enchytraeidae (Oligochaeta) in Greek agricultural soils. Soil Org. 81, 197–212 (2009).

278. Wang, Y. et al. Long-term impact of farming practices on soil organic carbon and nitrogen pools and microbial biomass and activity. Soil Tillage Res. 117, 8–16 (2011).

279. Wells, A. T., Chan, K. Y. & Cornish, P. S. Comparison of conventional and alternative vegetable farming systems on the properties of a yellow earth inNew South Wales. Agric. Ecosyst. Environ. 80, 47--60 (2000).

280. Welsh, C., Tenuta, M., Flaten, D. N., Thiessen-Martens, J. R. & Entz, M. H. High yielding organic crop management decreases plant-available but not recalcitrant soil phosphorus. Agron. J. 101, 1027–1035 (2009).

281. Domagała-Świątkiewicz, I. & Gąstoł, M. Soil chemical properties under organic and conventional crop management systems in south Poland. Biol. Agric. Hortic. 29, 12–28 (2013).
